# Isoquercitrin alleviates pirarubicin-induced cardiotoxicity *in vivo* and *in vitro* by inhibiting apoptosis through Phlpp1/AKT/Bcl-2 signaling pathway

**DOI:** 10.3389/fphar.2024.1315001

**Published:** 2024-03-18

**Authors:** Lei Wang, Jiulong Ma, Chen Chen, Bin Lin, Sicong Xie, Weiwei Yang, Jiajia Qian, Yang Zhang

**Affiliations:** ^1^ Department of Rehabilitation Medicine, School of Acupuncture-Moxibustion and Tuina and School of Health Preservation and Rehabilitation, Nanjing University of Chinese Medicine, Nanjing, China; ^2^ Department of Experimental Pharmacology and Toxicology, School of Pharmacy, Jilin University, Jilin, China; ^3^ Key Laboratory of Intelligent Pharmacy and Individualized Therapy of Huzhou, Department of Pharmacy, Changxing People’s Hospital, Huzhou, China

**Keywords:** isoquercitrin, Phlpp1/AKT/Bcl-2 signaling pathway, pirarubicin, apoptosis, oxidative damage

## Abstract

**Introduction:** Due to the cardiotoxicity of pirarubicin (THP), it is necessary to investigate new compounds for the treatment of THP-induced cardiotoxicity. Isoquercitrin (IQC) is a natural flavonoid with anti-oxidant and anti-apoptosis properties. Thus, the present study aimed to investigate the influence of IQC on preventing the THP-induced cardiotoxicity *in vivo* and *in vitro*.

**Methods:** The optimal concentration and time required for IQC to prevent THP-induced cardiomyocyte damage were determined by an MTT assay. The protective effect was further verified in H9c2 and HCM cells using dichlorodihydrofluorescein diacetate fluorescent probes, MitoTracker Red probe, enzyme-linked immunosorbent assay, JC-1 probe, and real time-quantitative polymerase chain reaction (RT-qPCR). Rats were administered THP to establish cardiotoxicity. An electrocardiogram (ECG) was performed, and cardiac hemodynamics, myocardial enzymes, oxidative stress indicators, and hematoxylin-eosin staining were studied. Voltage-dependent anion channel 1 (VDAC1), adenine nucleotide translocase 1 (ANT1), and cyclophilin D (CYPD) were detected by qRT-PCR, and the Phlpp1/AKT/Bcl-2 axis proteins were detected by western blot, confirming that IQC markedly increased cell viability and superoxide dismutase (SOD) levels, diminished the levels of ROS and MDA, and elevated mitochondrial function and apoptosis *in vivo* and *in vitro*.

**Results:** Results showed that IQC reduced THP-induced myocardial histopathological injury, electrocardiogram (ECG) abnormalities, and cardiac dysfunction *in vivo*. IQC also decreased serum levels of MDA, BNP, CK-MB, c-TnT, and LDH, while increasing levels of SOD and GSH. We also found that IQC significantly reduced VDAC1, ANT1, and CYPD mRNA expression. In addition, IQC controlled apoptosis by modulating Phlpp1/AKT/Bcl-2 signaling pathways. IQC markedly increased H9c2 and HCM cell viability and SOD levels, diminished the levels of ROS and MDA, and elevated mitochondrial function in H9c2 and HCM cells to defend against THP-induced cardiomyocyte apoptosis *in vitro*. The AKT inhibitor IMQ demonstrated that IQC lacked antioxidant and anti-apoptotic properties. Moreover, our data showed that IQC regulates Phlpp1 expression, thereby influencing the expression levels of p-AKT, cytochrome c, caspase-3, caspase-9, Bcl-2, and Bax.

**Discussion:** In conclusion, our results indicate that IQC protects the changes in mitochondrial membrane permeability in cardiomyocytes by regulating the Phlpp1/AKT/Bcl-2 signaling pathway, inhibits the release of cytc from the mitochondrial inner membrane to the cytoplasm, forms apoptotic bodies, induces cell apoptosis, and reduces THP induced cardiotoxicity.

## 1 Introduction

**FIGURE 1:**
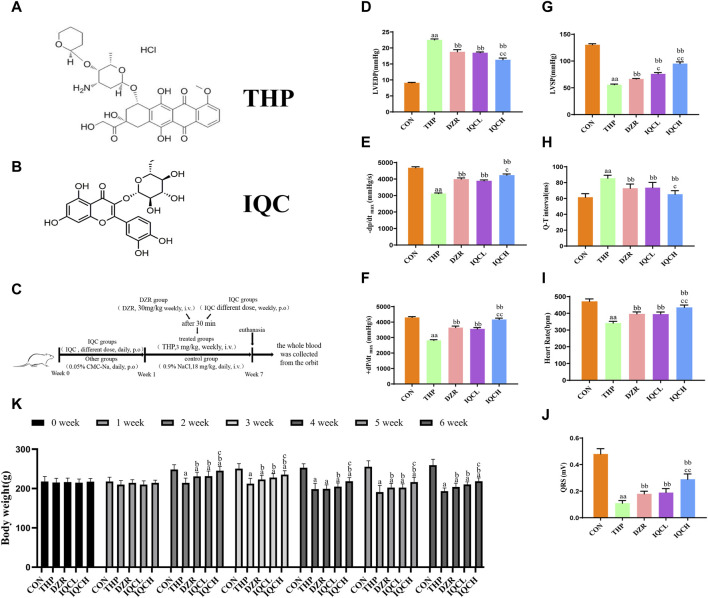
IQC’s effects on THP-induced hemodynamic indices and electrocardiograms in rats. **(A)** Chemical structure of THP. **(B)** Chemical structure of IQC. **(C)** Graphic summary of animal experiments. **(D)** Left ventricular end-diastolic pressure (LVEDP). **(E,F)** Maximal left ventricular pressure change (±dp/dtmax). **(G)** Left ventricular systolic pressure (LVSP). **(H)** Representative cardiac Q–T interval. **(I)** Representative heart rate. **(J)** Cardiac QRS complex. **(K)** Representative body weight. ^a^ *p* < 0.05 compared with the CON group; ^aa^ *p* < 0.01 compared with the CON group; ^b^ *p* < 0.05 compared with the THP group; ^bb^ *p* < 0.01 compared with the THP group; ^c^ *p* < 0.05 compared with the DZR group; ^cc^ *p* < 0.01 compared with the DZR group. Data were analysed using single-factor ANOVA followed by t-test. The data are expressed as the mean ± square error of the mean of three independent experiments.

Pirarubicin (THP, Figure 1A) is an anthracycline antitumour drug. It is widely used in the treatment of blood and solid tumours due to its high efficacy and broad spectrum (DeSantis et al., 2016); however, its long-term use causes severe myocardial damage. The severity of myocardial injury increases gradually as the cumulative dose of THP in the body increases (Varga et al., 2015; Gyöngyösi et al., 2020). When the cumulative THP concentration increases from 400 mg/m^2^ to 700 mg/m^2^, the incidence of heart failure rises from 5% to 48% (Lipshultz et al., 2005; Christiansen and Autschbach, 2006; Li and Hill, 2014). Currently, there are over 3.1 million breast cancer survivors in the United States with over 260,000 new cases projected annually (Padegimas et al., 2019). Anthracyclines can cause cardiotoxicity, manifested as left ventricular systolic dysfunction, in approximately 8% of patients (Titus et al., 2023). Therefore, about 21,000 breast cancer patients will suffer from cardiac toxicity due to the use of anthracycline drugs every year. Due to these adverse effects, THP has limited applicability despite its potency and effectiveness.

In recent years, a vast quantity of research has shown that THP-induced myocardial injury occurs via multiple mechanisms, including oxidative stress, mitochondrial damage and apoptosis (Lipshultz et al., 2008; Chen et al., 2015; Zhang et al., 2019). Oxidative stress is a key mechanism underlying the development of THP-induced myocardial injury. THP generates enormous quantities of reactive oxygen species (ROS), which then cause mitochondrial dysfunction and cellular apoptosis (Rochette et al., 2015; Damiani et al., 2016). Inhibition of oxidative stress may therefore be an effective preventative and therapeutic measure against THP-induced cardiotoxicity.

The mitochondria have emerged as important regulators of cardiovascular health and diseases (Dai et al., 2012; Marin et al., 2021). Anthracycline-induced myocardial damage is associated with disorders of the mitochondria. Upon entering cardiomyocytes, anthracyclines alter the permeability of the mitochondrial membrane and release mitochondrial cytochrome c (Cyt c) into the cytoplasm, thereby inducing apoptosis (Gorini et al., 2018). Simultaneously, anthracyclines can inhibit the activity of transcriptase in mitochondria, leading to continuous oxidative stress in damaged mitochondria and aggravating the occurrence of apoptosis. In addition, when mitochondria are damaged, the mitochondrial membrane potential and mitochondrial membrane permeability change. Mitochondrial membrane permeability is regulated by the mitochondrial membrane permeability transition pore (MPTP) (Dhingra et al., 2020). The MPTP is located between the inner and outer mitochondrial membranes. It is typically a channel composed of multiple proteins: outer membrane VDAC1, ANT1, and CYPD (Liu et al., 2016; Waseem and Wang, 2023).

Isoquercitrin (IQC, Figure 1B), a naturally occurring flavonoid abundant in medicinal and edible plants, possesses numerous biological properties, such as antioxidant and anti-inflammatory properties. IQC protects against renal ischaemia/reperfusion injury by reducing oxidative stress and inhibiting apoptosis (Liang et al., 2020). IQC administration provides significant protection against cisplatin-induced nephrotoxicity, presumably by reducing apoptosis, inflammation, and oxidative stress (Wang F. et al., 2020). IQC decreases lipopolysaccharide-induced cardiac dysfunction by inhibiting inflammatory responses and increasing fatty acid oxidation, in part by activating AMP-activated protein kinase (Huang et al., 2018). Nonetheless, the protective effect of IQC against THP-induced cardiotoxicity is still unknown.

Apoptosis plays an important role in the pathogenesis of THP induced cardiotoxicity by activating caspase (Zhang et al., 2019). Bcl-2 family proteins, such as Bcl-2, can inhibit cell apoptosis (Das and Steenbergen, 2012). When the expression of Bcl-2 decreases, cyt-c is released through the mitochondrial membrane, leading to the formation of apoptotic bodies (Alvarez et al., 2003). Apoptososomes activate effector caspase-3 instead. In addition, protein kinase B (AKT) is a key molecule in THP induced cell apoptosis. AKT phosphorylation can activate Bcl-2 and protect myocardial cells from apoptosis (Zhang X. et al., 2020). Further research has found that Phlpp1 can directly affect the phosphorylation of AKT, thereby affecting the occurrence of cell apoptosis (Newton and Trotman, 2014). Therefore, the purpose of this study was to investigate the protective effect of IQC against THP-induced cardiotoxicity and determine whether or not this effect was mediated by modulating Phlpp1/AKT/Bcl-2 signaling pathways, ultimately regulating mitochondrial damage-induced programmed apoptosis.

## 2 Materials and methods

### 2.1 Reagents

THP (C_32_H_37_NO_12_ 10 mg) was obtained from Shenzhen Wanle Pharmaceutical Co., Ltd. (Shenzhen, China). IQC (purity, ≥99%) was obtained from Nanjing Jingzhu Biological Technology Co., Ltd. (Nanjing, China). TRIzol reagent (Invitrogen, Carlsbad, CA, United States) were purchased from Thermo Fisher Scientific (Shanghai, China). GoldenTran-D reagent was purchased from Jin Chuan Technology Co., Ltd. (Changchun, China). Bicinchoninic acid (BCA) protein, 4′,6- diamidino-2-phenylindole (DAPI), Cell Mitochondria Isolation Kit (C3601), AKT inhibitor (SF2784), MitoTracker Red CMXRos (C1035), mitochondrial membrane potential assay kit with JC-1 (C2006), ROS assay kit (S0033S), calcein/propidium iodide (PI) cell viability/cytotoxicity assay kit (C2015M), and mitochondrial permeability transition pore assay kit (C2009S) were purchased from Beyotime Biotechnology Co., Ltd. (Shanghai, China).

Superoxide dismutase (SOD) and malondialdehyde (MDA) detection kits were obtained from Nanjing Jiancheng Institute of Biotechnology (Nanjing, China). TransScript^®^ All-in-One First-Strand cDNA Synthesis SuperMix for qPCR (One-Step gDNA Removal) and TransStart^®^ Top Green qPCR SuperMix were provided by Beijing TransGen Biotech Co., Ltd. (Beijing, China). Anti-cytochrome C (ab133504), anti-COX IV (ab202554), anti-Bcl-2 (ab182858), and anti-Bax (ab32503) were purchased from Abcam (Cambridge, MA, United States). Anti-Phlpp1(A9542), pan-AKT (A18120), Phospho-AKT1-S473+AKT2-S474+AKT3-S472 rabbit pAb (AP1068), anti-caspase-9 (A11910), anti-caspase-3 (A2156), active caspase-3 (A11021), horseradish peroxide (HRP) goat anti-rabbit IgG (H+L) (AS014), and HRP goat anti-mouse IgG (H+L) (AS003) were purchased from ABclonal Biotechnology Co., Ltd. (Wuhan, China). Anti-glyceraldehyde 3-phosphate dehydrogenase (GAPDH) was purchased from Affinity Biosciences (Jiangsu, China).

### 2.2 Animals and experimental protocol

This research was approved by the Animal Care and Ethics Committee of Jilin University (Changchun, China; Grant no. 20170503) and conducted in accordance with the National Institutes of Health’s Guide for the Care and Use of Laboratory Animals. The Animal Experiment Center of Jilin University supplied 40 male Wistar rodents of clean quality, weighed 200 ± 20 g, of a clean grade. The rodents were confined in a standard environment with a temperature of 22°C ± 3°C, a humidity of 50% ± 10%, and 12 h of light/dark cycles. All test animals were euthanized by inhaling CO_2_ at a rate of 30% volume displacement per minute.

According to our previous reports, the dosage of THP has been determined (Zhang et al., 2023). Based on previous research reports (Sumi et al., 2013; Huang et al., 2018), we have determined the dosing concentration of IQC. After adaptive breeding for 1 week, the rats were randomly divided into five groups (*n* = 8): 1) Control (CON), 2) THP (3 mg/kg/weekly), 3) THP + Dexrazoxane (DZR) (30 mg/kg/weekly), 4) THP + low-dose IQCL (50 mg/kg/d), and 5) THP + high-dose IQCH (100 mg/kg/d). IQC-treated rats were given varying doses of IQC every day for a week before receiving THP injections. For a week, THP and DZR-treated rats were given sodium carboxymethylcellulose (CMC-Na) via gavage. In addition, a weekly injection of 3 mg/kg THP was administered via caudal vein for 6 weeks in order to develop the cardiotoxicity model. Rats were subsequently administered DZR (30 mg/kg) intraperitoneally once per week for 6 weeks. In the control group, rats were administered CMC-Na via gavage for 7 days, followed by saline via injection into the caudal vein for 6 weeks (Figure 1C).

### 2.3 Detection of hemodynamic indexes and electrocardiogram

Rats were rendered unconscious by intraperitoneal injection of sodium pentobarbital. After severing the right common carotid artery, a 1 mm-diameter plastic cannula was inserted into the left ventricle of rodents and affixed to the BL-420E biological function experiment system. We measured the left ventricular systolic pressure (LVSP), end-diastolic pressure (LVEDP), and maximal rate of change in left ventricular pressure (±dp/dtmax). The ECG of a rat was analyzed utilizing the conventional limb II.

### 2.4 Assessment of biochemical parameters

Blood samples were drawn from the abdominal aorta and centrifuged at 3,000 rpm for 15 min following 6 h of standing. Using ELISA detection kits, the serum concentrations of BNP, CK-MB, c-TnT, MDA, LDH, GSH and SOD were then measured, per the manufacturer’s instructions. In short, the ELISA kit used in this study is a double antibody sandwich ELISA method. Antibodies are coated on an enzyme-linked immunosorbent assay (ELISA) plate, and antigens in the sample or standard will bind to the coated antibodies during the experiment. Add otinylated antibodies and horseradish peroxidase labeled avidins in sequence. Biotinylated antibodies bind to antigens bound to coated antibodies, and biotin specifically binds to avidin to form immune complexes. By adding a chromogenic substrate (TMB), TMB appears blue under the catalysis of horseradish peroxidase, and turns yellow after adding a termination solution. Measure the OD value at a wavelength of 450 nm using an enzyme-linked immunosorbent assay (ELISA) reader. The concentration of the detected substance is directly proportional to the OD450 value. Calculate the concentration of the relevant substance in the sample by plotting a standard curve.

### 2.5 Hematoxylin-eosin (HE) staining

Before paraffin embedding, 10% formalin was utilized to cure the cardiac samples. The paraffin segments of each group were stained with HE solution. HE-stained images were viewed at ×200 magnification using a Nikon Eclipse 80i microscope (Nikon, Chiyoda, Japan).

### 2.6 Tissue mitochondria extraction

Cut myocardial tissue weighing about 100 mg, rinse once with PBS, cut the tissue into fine pieces with scissors; add 10 volumes of pre-cooled PBS, ice bath for 3 min; subsequently, centrifuge at 10,000 rpm for 20 s, discard the supernatant; add 8 times the volume of pre-cooled trypsin digestion solution, ice bath for 20 min; after digestion, centrifuge at 10,000 rpm for 20 s, discard the supernatant; add 2 times the volume of the corresponding mitochondrial separation reagent, resuspend the tissue; centrifuge at 10,000 rpm for 20 s, discard the supernatant; add 8 times the volume of pre-cooled mitochondrial separation reagent, homogenize in ice bath, centrifuge the homogenate at 5,000 rpm, 4°C for 5 min; transfer the supernatant to another centrifuge tube, centrifuge at 15,000 rpm, 4°C for 10 min; collect the supernatant, the supernatant is the cell pulp with mitochondria removed protein, and the precipitate is the isolated mitochondria.

### 2.7 qRT-PCR assay

Total RNA was extracted using TransScript All-in-One First-Stand cDNA Synthesis SuperMix. First-strand complementary DNA synthesis was performed using the reverse transcription system kit according to the manufacturer’s instructions. The primer sequences are listed in Table 1. The ratios of the relative expressions of target genes to those of the housekeeping genes were calculated using the 2^−ΔΔT^ method. All reactions were performed in triplicates for each sample.

**TABLE 1:** Primer sequences for Real-time PCR.

Genes	Sequence (5′-3′)	Reference sequence
*Rattus VDAC1*	F: GTT​GGG​GAT​GCG​AGA​GTT​GA	NM_031353.1
	R: GGA​ATG​GGG​TTT​CCG​CTG​TA	
*Rattus ANT1*	F: CCA​AGC​TCT​CAA​CTT​CGC​CT	NM_053515.1
	R: GCG​CCA​GAA​CTG​CTT​ATG​AC	
*Rattus CYPD*	F: GGC​ACT​TGT​GTC​CTG​CTT​TC	NM_001004279.1
	R: AAT​TCG​TCC​AAC​TCG​CTC​CC	
*Rattus GAPDH*	F: AGT​GCC​AGC​CTC​GTC​TCA​TA	NM_017008.4
	R: ATC​CGT​TCA​CAC​CGA​CCT​TC	
*Homo VDAC1*	F: ATC​ACA​TGG​TGA​CAA​CAC​TCA​GA	NM_003374.3
	R: AGA​CAA​CAG​AAG​AAG​GAT​GAG​GTT​T	
*Homo ANT1*	F: GTT​CCT​CAC​CGC​AGC​TAC​TT	NM_001151.4
	R: CAA​TGA​TGG​TAT​GGC​GTG​CG	
*Homo CYPD*	F: TCA​AGA​TGT​CGC​ACC​CGT​C	NM_005038.3
	R: AGG​TTT​CCC​AGT​CGT​GTG​TC	
*Homo GAPDH*	F: TCG​GAG​TCA​ACG​GAT​TTG​GT	NM_002046.7
	R: TTC​CCG​TTC​TCA​GCC​TTG​AC	
*Rattus COXⅣ*	F: GTA​CCG​CAT​CCA​GTT​TAA​CGA​GAG	NM_017202.1
	R: CGC​AGT​GAA​GCC​GAT​GAA​GAA​C	
*Homo COXⅣ*	F: CAG​CTT​ATA​TGG​ATC​GGC​GTG​AC	NM_001318786.3
	R: GCC​TTC​TCC​TTC​TCC​TTC​AAT​GC	

### 2.8 Western blot assay

A radioimmunoprecipitation assay lysate buffer comprising protease and phosphatase inhibitors was used to homogenize whole protein samples from cells or myocardial tissue. Using a BCA protein assay reagent, the protein concentration of each sample was determined. The proteins were separated by SDS gel (8%–12%) and then transferred to PVDF membranes (Massachusetts, United States). Blots were blocked in 5% milk and incubated with primary antibodies at 4°C for a single night (Table 2). The subsequent step involved incubation with a secondary antibody (Table 2). Bands on blots were visualized using chemiluminescence and quantified using Image J software version 1.42q (National Institutes of Health, Bethesda, Maryland, United States).

**TABLE 2:** Antibodies used in Western blot analysis.

Antibody	Dilutions	Source	Company
Primary antibodies			
Phlpp1	1:1,000	Rabbit	ABclonal, Wuhan, China
Pan-AKT	1:1,000	Rabbit	Abcam, United States
Phospho-AKT1	1:1,000	Rabbit	ABclonal, Wuhan, China
Cytochrome C	1:1,000	Rabbit	Abcam, United States
COX IV	1:1,000	Rabbit	Abcam, United States
Caspase-9	1:1,000	Rabbit	ABclonal, Wuhan, China
Caspase-3	1:1,000	Rabbit	ABclonal, Wuhan, China
Cleaved-caspase-3	1:1,000	Rabbit	ABclonal, Wuhan, China
Bax	1:1,000	Rabbit	Abcam, United States
Bcl-2	1:1,000	Rabbit	Abcam, United States
Secondary antibody			
HRP Goat anti-Rabbit IgG-	1:5,000	Goat	ABclonal, Wuhan, China

### 2.9 Cytotoxicity of IQC

The American Type Culture Collection provided access to rat cardiac myocytes (H9c2). From ScienCell Research Laboratories, human cardiac myocytes (HCM) were obtained. H9c2 cells were grown in DMEM (with 4.5 mg/mL of glucose) supplemented with 10% foetal bovine serum. In cardiac myocyte medium, HCM cells were cultured. The cells were cultured in 5% CO_2_ at 37°C, and the medium was replaced every 2 to 3 days.

Prior to being treated with various concentrations of IQC (5, 10, 25, 50, 70, 90, 100, 200, and 500 μM), 5 × 10^4^ cells/mL were seeded in 96-well plates. Equal amounts of serum-free medium were used to treat the cells in the control (CON) group. In addition, cell viability was determined using the MTT assay after 6, 12, 24, and 36 h of incubation. Briefly, MTT (5 mg/mL, 10 μL) solution was added to each plate, incubated for 4 h at 37°C, and then the medium was discarded. Finally, 150 μL of dimethyl sulphoxide was added to dissolve the formazan crystals, and the absorbance was measured at 560 nm using a BioRad POLARstar OPTIMA multi-detection microplate reader (San Diego, California, United States).

### 2.10 IQC inhibited THP-induced cell injury

H9c2 and HCM cells were inoculated at a density of 5 × 10^4^ cells/mL in 96-well plates for 6, 12, 24, and 36 h and then treated with various concentrations of THP (1, 3, 5, 7, and 9 μM). In the CON group, cells were treated with equal quantities of serum-free medium. Lastly, cell viability was determined using the MTT assay as described previously.

### 2.11 Cell treatments

The cells were subsequently separated into five groups. Cells in the CON group were cultured in serum-free DMEM high-glycaemic culture medium; cells in the THP group were cultured in serum-free DMEM high-glycaemic culture medium containing 5 μM of THP; Cells in the IQC group were treated with 70 µM of IQC 30 min before administering 5 μM of THP, and cells in the AKT inhibitor (Imidazoquinoxaline [IMQ]) group were treated with 1 μM of IMQ 30 min before treatment with 5 μM THP. 30 min prior to administering 5 µM of THP, serum-free DMEM high-glycemic culture medium containing 70 µM IQC and 1 µM IMQ was added to the IQC+IMQ group. Each group of cells was incubated for 24 h.

### 2.12 Mesurement of SOD and MDA levels

The cells were seeded at a density of 5 × 10^4^ cells/mL in 6-well culture plates and treated with various solutions before being incubated with THP for 24 h. Using commercial products according to the manufacturer’s instructions, the SOD and MDA levels in the cells were then measured.

### 2.13 Detection of intracellular ROS using a DCFH-DA probe

The cells were seeded at a density of 5 × 10^4^ cells/mL in 6-well culture plates and treated with various solutions before being incubated with THP for 24 h. After removing the medium, DCFH-DA (10 μM, 1.5 mL) was added to the wells and incubated for 30 min at 37°C. The cells were then fixed with 4% paraformaldehyde solution, stored at room temperature, and shielded from light for 10 min. Following this, the fixative was removed, the cells were rinsed twice with PBS, stained with DAPI, and then covered with the sample. After 5 min at room temperature, the DAPI staining solution was aspirated and the samples were observed using an Olympus (Tokyo, Japan) fluorescence microscope.

### 2.14 Detecting apoptosis via TUNEL

The cells were plated in 6-well culture plates at a density of 5 × 10^4^ cells/mL and treated with different solutions before incubation with THP for 24 h. Then, the cells were fixed with 4% paraformaldehyde, incubated with proteinase K solution for 5 min, rinsed twice with PBS, and incubated with Alexa Fluor 640-12-dUTP Labelling Mix for 60 min. The nuclei were stained with DAPI and observed by taking pictures with an inverted fluorescence microscope. Intact nuclei stained blue while apoptotic cells-stained red.

### 2.15 Cell viability detection using a calcein/propidium iodide assay kit

According to the experimental design, the cells were inoculated in a 6-well plate and then treated. The mixture was then incubated for 30 min in the dark at 37°C with 1 mL of calcein acetoxymethyl ester (AM)/PI detection working solution. The staining effect was observed under a fluorescence microscope following incubation (calcein-AM exhibits a green fluorescence, excitation-emission maxima [Ex/Em] = 494/517 nm; PI exhibits a red fluorescence, Ex/Em = 535/627 nm).

### 2.16 Detecting the cell mitochondrial membrane potential using a JC-1 probe

Upon completion of the cell treatment, the culture solution was removed, the cells were rinsed once with PBS, 1 mL of cell culture solution was added, 1 mL of JC-1 staining working solution was added, and the mixture was thoroughly stirred. The mixture was then incubated for 20 min at 37°C. The cells were rinsed twice with JC-1 staining buffer, 2 mL of cell culture medium was added, the cells were observed and photographed under a fluorescence microscope, and the intracellular mitochondrial membrane potential was determined.

### 2.17 Detecting the cell mitochondrial activity using a MitoTracker red CMXRos probe

After grouping and processing the cells, MitoTracker Red CMXRos working solution was added and incubated at 37°C for 15 min. After incubation, the cell culture medium was added, washed twice, fixed with 4% paraformaldehyde solution for 10 min, stained with DAPI solution, and finally observed and imaged under a fluorescence microscope to analyse the activity of mitochondria in the cell.

### 2.18 Mitochondrial permeability transition pore

The calcein-AM release test was used to detect the opening level of MPTP in cardiomyocytes. After grouping and processing the cells, an appropriate volume of calcein-AM/cobalt (II) chloride (COCl_2_) staining solution was added and shaken gently to ensure that the dye evenly covered all cells. The mixture was then incubated at 37°C in the dark for 30 min. After incubation, the culture medium was replaced with a fresh culture medium preheated to 37°C and incubated for 30 min. The culture medium was aspirated and observed under a fluorescence microscope.

### 2.19 Mitochondrial function assessment by Seahorse analyzer

H9c2 and HCM cells were seeded into Seahorse XFe24 V7 microplates at a density of 5 × 10^3^ cells/well, followed by treatment with different compounds for 24 h. And the culture medium was substituted with Agilent Seahorse XF DMEM medium supplemented with 1 mM of sodium pyruvate, 2 mM of glutamine and 1 mM D-glucose. The cells were then placed in an incubator at 37°C for 60 min prior to test, followed by washing with Seahorse buffer for subsequent experimental operations. Meanwhile, 1.5 μM oligomycin and 0.5 μM rotenone/antimycin A (Rot/AA) were injected to assess the real-time ATP rate. While oligomycin (1.5 μM), FCCP (1.0 μM) and Rot/AA (0.5 μM) were automatically injected to measure the oxygen consumption rate (OCR). Protein concentrations were determined by BCA protein assay kit and the real time ATP rate and OCR were normalized by the total protein. The temperature of all above experimental procedures was maintained at 37°C.

### 2.20 Cell mitochondria extraction

To extract cellular mitochondria, the cells were grouped and processed, washed with PBS three times, and trypsinised, after which we collected the cells at 3,000 rpm, centrifuged the mixture at room temperature for 10 min, and retained the cell pellets. The cell pellets were gently resuspended in ice-cold PBS and a small number of cells was extracted for counting. The remaining cells were centrifuged at 4°C and 6,000 rpm for 5 min to obtain cell pellets. We then discarded the supernatant, added a mitochondrial separation reagent to approximately 2 × 10^7^ cells, and placed the mixture on ice for 15 min. The cell suspension was then transferred to a glass homogeniser and homogenised at 20°C. We centrifuged the cell homogenate at 4°C, 8,000 rpm for 10 min, and transferred the supernatant to another centrifuge for centrifugation at 4°C, 13,000 rpm for 10 min, yielding a precipitant containing the resulting cell mitochondria. The supernatant was a cytoplasmic protein from which the mitochondria had been removed.

### 2.21 Network pharmacology analysis

From the PharmMapper database, putative IQC-related targets were extracted. Comparative Toxicogenomics Database was searched with the keyword “Pirarubicin cardiotoxicity” to identify anthracyclines-related targets. The IQC-related and Anthracyclines-related targets were then analyzed using an online Venn diagram tool. The protein-protein interaction (PPI) network of obtained intersection targets was constructed utilizing the STRING database and the Cytoscape program.

### 2.22 Molecular docking assay

Download the 3D structure in SDF format from PubChem data according to the CAS number of the small molecule, import the structure into ChemBio3D Ultra 14.0 to minimize the energy, set Minimum RMS Gradient to: 0.001. The molecule is saved in mol2 format. Import the optimized small molecules into AutodockTools-1.5.6 for hydrogenation, calculating the charge, distributing the charge, setting the rotating key and saving it in the “pdbqt” format.

Download the protein structure of AKT1 (PDB ID: 1UNQ) from the PDB database, download the protein structure of Phlpp1 (ID: O60346) from the Uniprot database, and import the protein structure into Pymol 2.3.0 to remove protein crystallized water, original ligands, etc. The protein structure is imported into AutoDocktools (v1.5.6) for hydrogenation, calculation of charge, distribution of charge, specifying the atomic type and saving it in the “pdbqt” format.

Use POCASA 1.1 to predict the protein binding site, use AutoDock Vina1.1.2 for docking, and the AKT1 related parameters are set to: center_x = 14.7, center_y = 21.9, center_z = 16.6; Search space: size_x:50, size_y: 50, size_z:50 (the spacing of each lattice is 0.375Å), exhaustiveness:10, the remaining parameters are default settings; Phlpp1 related parameter settings For: center_x = −3.1, center_y = −6.0, center_z = 2.6; search space: size_x: 50, size_y: 50, size_z: 50 (the spacing of each grid is 0.375Å), exhaustiveness: 10, the remaining parameters are the default settings. PyMOL2.3.0 and Ligplot V2.2.5 are used to analyze the interaction pattern of the docking results.

### 2.23 Statistical analysis

The SPSS software program (version 20.0; SPSS Inc., Chicago, IL, United States) was used to perform the statistical analysis. Data were presented using single-factor ANOVA followed by t-test. All data are presented as the mean ± SD. Differences were statistically significant at *p* < 0.05 and highly significant at *p* < 0.01.

## 3 Results

### 3.1 Effects of IQC on THP-induced cardiotoxicity hemodynamics and electrocardiograph

Compared to the CON group, the THP group had substantially reduced LVSP and ±dp/dtmax values, but significantly higher LVEDP values. The DZR and IQC groups have lower LVEDP values than the THP group, while LVSP and ±dp/tmax have significantly increased. Compared to the DZR group, the LVSP levels of rats in the IQCL group increased significantly; the LVEDP levels of rats in the IQCH group decreased significantly, while LVSP and ±dp/dtmax increased significantly (Figures 1D–G).

Compared to the CON group, the QRS complex voltage in the THP group was significantly lower, their Q-T interval was significantly longer, and their heart rate (HR) was significantly diminished. Comparatively to the THP group, the QRS complex voltage and HR of rodents in the DZR and IQC groups increased significantly, whereas the Q-T interval decreased. IQCH outperformed DZR on a variety of electrocardiogram parameters (Figures 1H–J).

### 3.2 IQC ameliorated rat body weight

As shown in Figure 1K, rodents in the THP group experience significant weight loss relative to rats in the control group. Compared to the THP group, rodents in the IQCL and IQCH groups gained significantly more weight. In terms of alleviating weight loss, the IQCH group performed better than the DZR group, whereas the IQCL group did not differ significantly from the DZR group.

### 3.3 Effects of IQC on THP-induced cardiotoxicity myocardial enzymes

**FIGURE 2:**
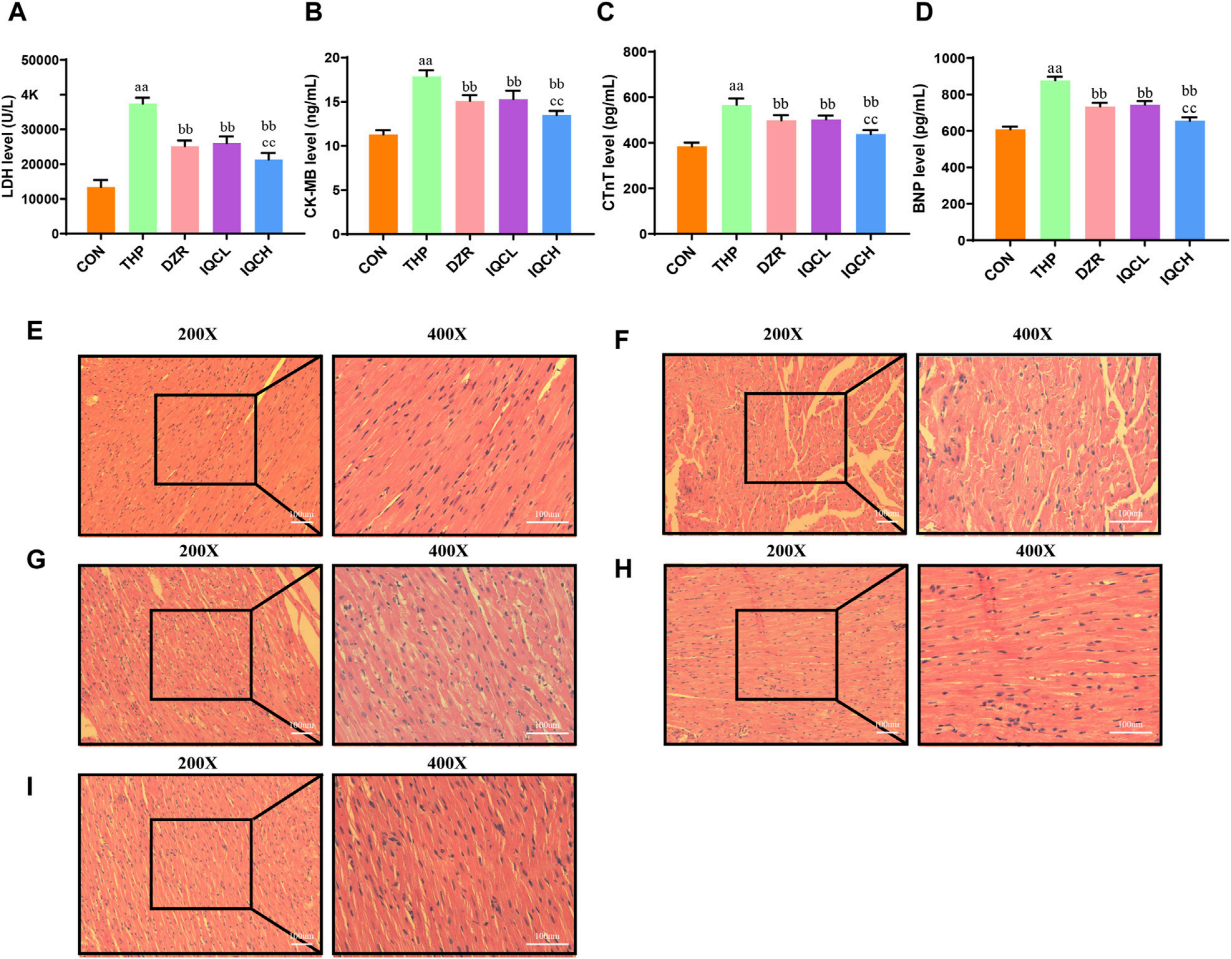
Effects of IQC on rat cardiac serum cardiac enzymes and histological alterations produced by THP. **(A)** Representative levels of lactate dehydrogenase (LDH). **(B)** Representative levels of creatine kinase MB (CK-MB). **(C)** Representative cardiac troponin T levels (c-TnT). **(D)** Representative brain natriuretic peptide levels (BNP). **(E–I)** Staining using H&E (200X, 400X). ^a^ *p* < 0.05 compared with the CON group; ^aa^ *p* < 0.01 compared with the CON group; ^b^ *p* < 0.05 compared with the THP group; ^bb^ *p* < 0.01 compared with the THP group; ^c^ *p* < 0.05 compared with the DZR group; ^cc^ *p* < 0.01 compared with the DZR group. Data were analysed using single-factor ANOVA followed by t-test. The data are expressed as the mean ± square error of the mean of three independent experiments.

The serum levels of LDH, CKMB, c-TnT, and BNP are significantly higher in the THP group than in the CON group. DZR and IQC rats had substantially lower levels of LDH, CKMB, c-TnT, and BNP than THP rats. IQCH rats exhibit a greater decrease in LDH, c-TnT, and BNP levels than DZR rats (Figures 2A–D).

### 3.4 Effect of IQC on THP-induced cardiotoxicity myocardial histopathological change

The pathological alterations of myocardial tissue are depicted in Figures 2E–I. HE staining reveals that the myocardial tissue of the CON group is normal, with no visible hypertrophy or infiltration of inflammatory cells. Following treatment with THP, rat myocardial fibers are arranged irregularly, myocardial cells are edematous, gaps are substantially dilated, and cytoplasmic lysis can result in vacuolar degeneration and inflammatory cell infiltration. All of these alterations suggest severe myocardial injury. After IQC and DZR intervention, the intercellular space is reduced, the arrangement becomes more regular, and dispersed vacuole-like degeneration is diminished.

### 3.5 Effects of IQC on THP-induced cardiotoxicity oxidative stress in rats heart tissue

**FIGURE 3:**
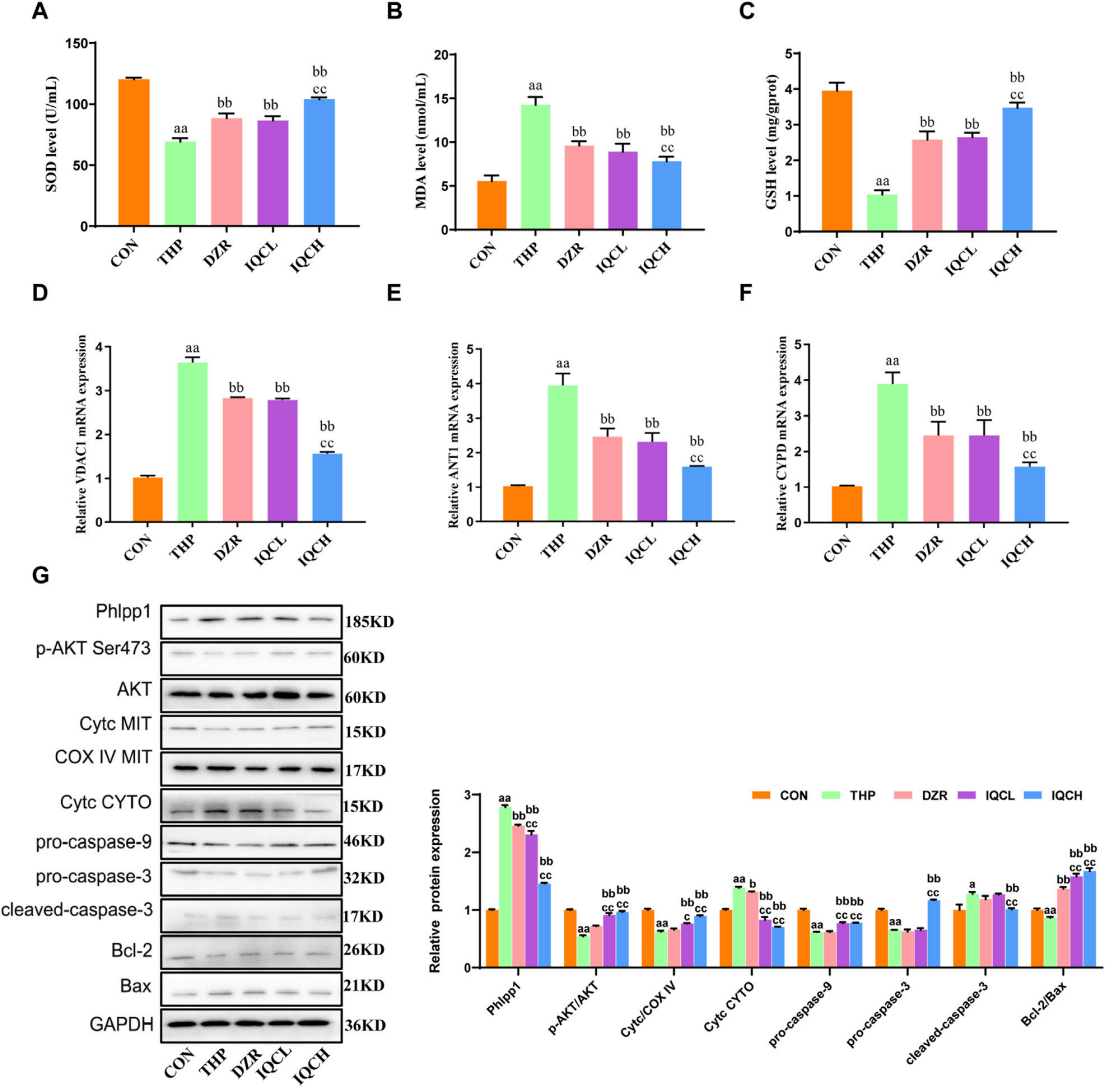
IQC reduced THP-induced cardiomyocyte apoptosis and oxidative stress in rats. **(A–C)** Representative superoxide dismutase (SOD), malondialdehyde (MDA) and glutathione (GSH) levels. **(D–F)** Representative VDAC1, ANT1, and CYPD mRNA expression levels. **(G)** Representative Phlpp1, Akt, Bcl-2, Bax, Cyt c and caspase protein expression levels in the cytoplasm or mitochondria; GAPDH or COX IV was used as an internal control. ^a^ *p* < 0.05 compared with the CON group; ^aa^ *p* < 0.01 compared with the CON group; ^b^ *p* < 0.05 compared with the THP group; ^bb^ *p* < 0.01 compared with the THP group; ^c^ *p* < 0.05 compared with the IMQ group; ^cc^ *p* < 0.01 compared with the IMQ group; ^d^ *p* < 0.05 compared with the IQC+IMQ group; ^dd^ *p* < 0.01 compared with the IQC+IMQ group. Data were analysed using single-factor ANOVA followed by t-test. The data are expressed as the mean ± square error of the mean of three independent experiments.

Compared to the CON group, SOD and GSH levels were reduced in the THP group, whereas MDA levels were higher (Figures 3A–C). Further analysis revealed that SOD and GSH levels in the IQC group were substantially higher than in the THP group, while MDA levels were significantly lower.

### 3.6 IQC reduces the expression levels of VDAC1, ANT1, and CYPD mRNA

Several studies have verified that THP can alter the permeability of mitochondrial membranes. VDAC1, ANT1, and CYPD are abundantly present in mitochondrial membranes. Therefore, we determined the mRNA expression levels of VDAC1, ANT1, and CYPD to confirm membrane alterations. VDAC1, ANT1, and CYPD mRNA expression was significantly elevated in rats with cardiactoxicity compared to rats in the CON group. As shown in Figures 3D–F, the mRNA levels of these genes were substantially lower in the IQCL, IQCH, and DZR-treated groups than in the cardiac toxicity model group. Specifically, at a dose of 100 mg/kg, IQC treatment significantly reduced the expression of the aforementioned mRNA.

### 3.7 Effect of IQC on Phlpp1/AKT/Bcl-2 signaling pathway in rats heart tissue

THP significantly diminished the levels of p-AKT/AKT and mitochondrial Cyt c, pro-caspase-9, pro-caspase-3, and Bcl-2/Bax, while significantly increasing the levels of Phlpp1, Cyt c, and cleaved-caspase-3 in the cytosol. IQC significantly increased the expression levels of p-AKT/AKT and mitochondrial Cyt c, as well as pro-caspase-9, pro-caspase-3, and Bcl-2/Bax, and decreased the expression levels of Phlpp1, cytosolic Cyt c, and cleaved-caspase-3 in comparison to the THP group (Figure 3G).

### 3.8 IQC reduced THP-induced H9c2 and HCM cellular damage

**FIGURE 4:**
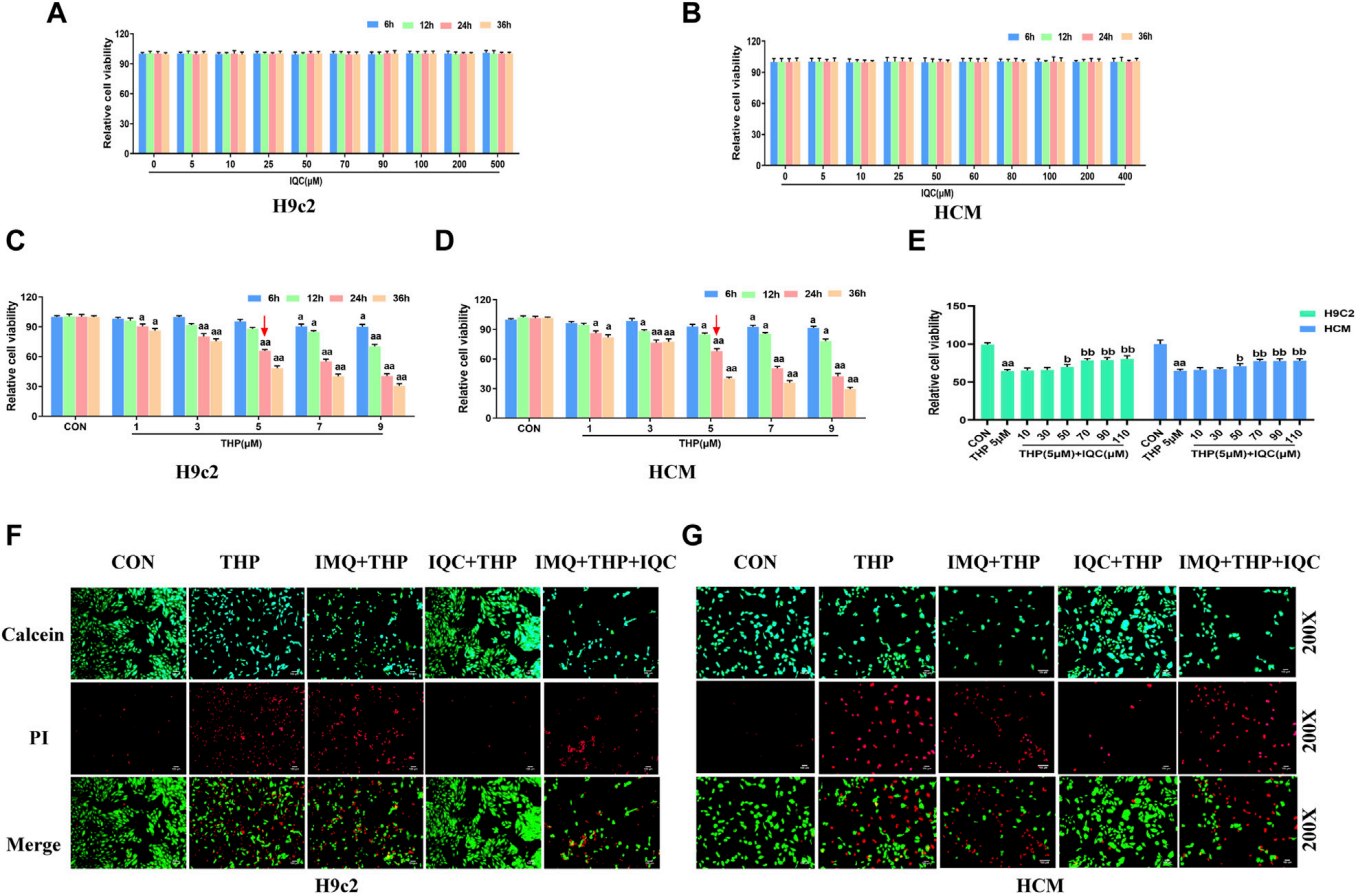
IQC protected H9c2 and HCM cells against THP-induced cardiotoxicity. **(A,B)** Cytotoxic effect of IQC on H9c2 and HCM cells. ^a^ *p* < 0.05 compared with the 0 μM group; ^aa^ *p* < 0.01 compared with the 0 μM group. **(C,D)** Effect of THP on the survival rate of H9c2 and HCM cells. ^a^ *p* < 0.05 compared with the CON group; ^aa^ *p* < 0.01 compared with the CON group. **(E)** Effects of IQC on the viability of H9c2 and HCM cells induced by THP. ^a^ *p* < 0.05 compared with the CON group; ^aa^ *p* < 0.01 compared with the CON group; ^b^ *p* < 0.05 compared with the THP group; ^bb^ *p* < 0.01 compared with the THP group. **(F,G)** Cell viability and cytotoxicity were evaluated using the calcein AM/PI Assay. Data were analysed using single-factor ANOVA followed by t-test. The data are expressed as the mean ± square error of the mean of three independent experiments.

We first incubated H9c2 and HCM cells with varying concentrations of IQC (5∼500 μM) for 6, 12, 24, and 36 h, and then used the MTT assay to determine whether IQC caused cytotoxicity. At 6, 12, 24, and 36 h, the cell viability in each group did not change substantially, indicating that IQC had no toxic effect on H9c2 and HCM cells (Figures 4A, B). We continued to determine the optimal concentration and time for H9c2 and HCM cells to sustain THP-induced injury. Compared to the CON group, the cell survival rates at various time points after treatment with 1 μM and 3 μM of THP were greater than 70%. In addition, treatment with 7 and 9 μM of THP for 6 and 12 h resulted in a higher than 70% cell survival rate. After 24 and 36 h, the cell survival rate was below 50%, which is unacceptable. The survival rate of cells treated with 5 μM of THP for 24 h was greater than 60%, whereas the survival rate of cells treated with 5 μM of THP for 36 h was unacceptable at less than 50%. The optimal concentration and time for the H9c2 and HCM cell injury models induced by THP were determined to be 5 μM and 24 h, respectively (Figures 4C, D).

In addition, the viability of H9c2 and HCM cells treated with 5 μM THP was significantly less than that of the CON group. Nonetheless, IQC at dosages of 70, 90, and 110 μM significantly increased the viability of H9c2 and HCM cells relative to the THP group (Figure 4E). Therefore, we determined that a 70 μM IQC concentration applied for 24 h optimally mitigated the THP-induced damage in H9c2 and HCM cells.

Using the calcein-AM/PI reagent, we continued to evaluate apoptosis (Figures 4F, G). The number of PI-positive cardiomyocytes significantly increased in the THP group compared to the CON group. Compared to the THP group, the number of PI-positive cardiomyocytes increased substantially in the IMQ group, but decreased considerably after IQC treatment. Comparatively, the IMQ+IQC group had fewer PI-positive cells than the IMQ group. IQC treatment substantially decreased the number of PI-positive cells and the apoptotic rate compared to the IMQ+IQC group.

### 3.9 IQC reduced THP-induced apoptosis

**FIGURE 5:**
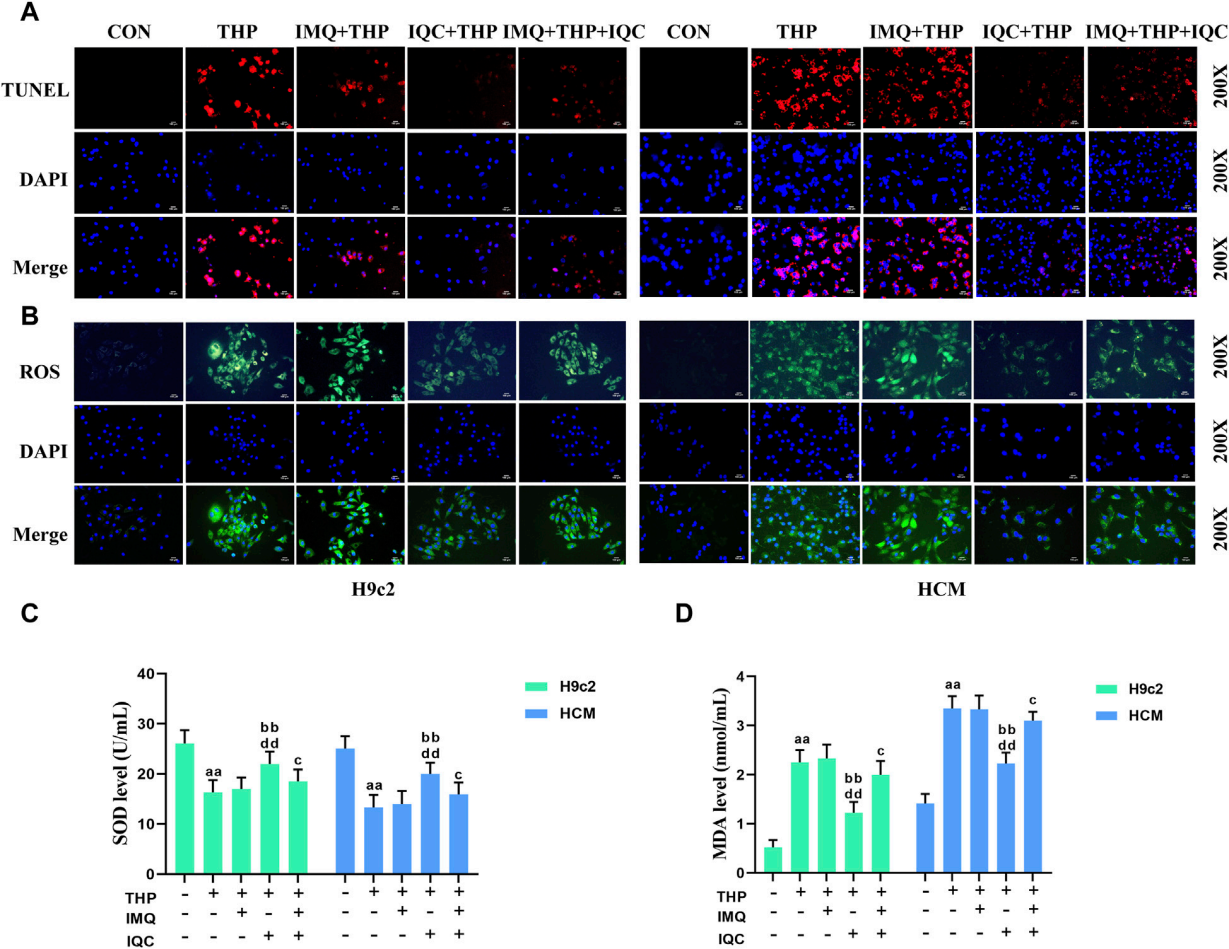
IQC reduced THP-induced cardiomyocyte apoptosis and oxidative stress. **(A)** Cell apoptosis was evaluated using the TUNEL assay. **(B)** Intracellular ROS levels were evaluated using the DCFH-DA staining assay. **(C,D)** Representative superoxide dismutase (SOD) and malondialdehyde (MDA) levels. ^a^ *p* < 0.05 compared with the CON group; ^aa^ *p* < 0.01 compared with the CON group; ^b^ *p* < 0.05 compared with the THP group; ^bb^ *p* < 0.01 compared with the THP group; ^c^ *p* < 0.05 compared with the IMQ group; ^cc^ *p* < 0.01 compared with the IMQ group; ^d^ *p* < 0.05 compared with the IQC+IMQ group; ^dd^ *p* < 0.01 compared with the IQC+IMQ group. Data were analysed using single-factor ANOVA followed by t-test. The data are expressed as the mean ± square error of the mean of three independent experiments.

To further investigate the effect of IQC on THP-induced apoptosis, we used a TUNEL assay kit (Figure 5A). The apoptotic rate of the THP group increased significantly compared with that of the CON group. Compared with the THP group, the IMQ group showed an increased rate of apoptosis, while that in the IQC group was significantly reduced. Compared with the IMQ group, the apoptotic rate of the IMQ+IQC group was reduced, whereas that of the IQC group was significantly reduced, indicating that AKT inhibitors can partially block IQC reduce the increase in apoptotic rate induced by THP.

### 3.10 IQC reduced THP-induced oxidative stress

THP substantially increased ROS and MDA levels while decreasing SOD levels in H9c2 and HCM cells (Figures 5B–D). Compared to the THP group, IMQ significantly increased ROS and MDA levels while decreasing SOD levels, while IQC significantly decreased ROS and MDA levels while increasing SOD levels. Compared to the IMQ group, the IMQ+IQC group had lower ROS and MDA levels, and higher SOD levels. Compared to the IMQ+IQC group, the IQC group exhibited a substantial decrease in ROS and MDA levels, but an increase in SOD levels.

### 3.11 IQC promotes mitochondrial energy metabolism in H9c2 and HCM cells

To further understand the mechanism responsible for the protective effects of IQC, mitochondrial energy metabolism and respiratory function in H9c2 and HCM cells was measured using the Agilent Seahorse XFp Cell Mito Stress Test. As expected, mitochondrial oxidative phosphorylation, as measured by OCR, was reduced in the THP group compared to the CON group. In addition, OCR was significantly higher in the IQC group compared to the THP group (Supplementary Figures S2A, C). Moreover, THP significantly decreased basal respiration, maximal respiration, ATP-associated OCR and proton leak levels (Supplementary Figures S2B, D) in H9c2 and HCM cells compared to the CON group. IQC attenuated these effects and significantly elevated basal respiration, maximal respiration, ATP-associated OCR and proton leak levels, which were strongly inhibited by THP. In addition, the addition of IMQ alone significantly reduced intracellular basal respiration, maximum respiration, ATP-associated OCR and proton leak levels. When IMQ was added to IQC, its improvement effect was also significantly reduced.

### 3.12 IQC restored THP-induced mitochondrial activity among H9c2 and HCM cells

**FIGURE 6:**
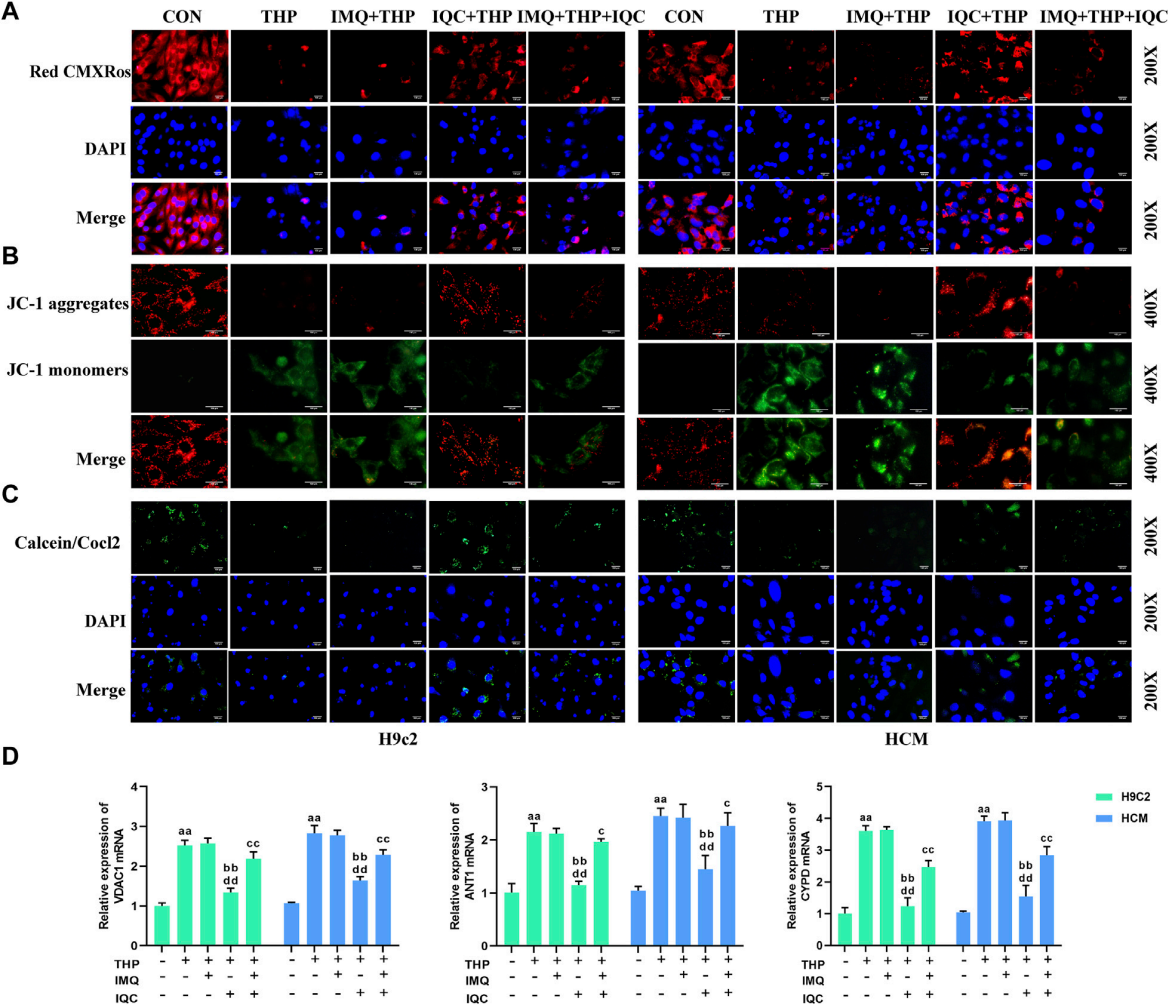
IQC ameliorated THP-induced cardiomyocyte mitochondrial activity and mitochondrial membrane gene expression. **(A)** Mitochondrial activity was evaluated using the MitoTracker Red CMXRos Assay. **(B)** Mitochondrial membrane potential was evaluated using the JC-1 assay. **(C)** Cell mitochondrial membrane permeability was evaluated using the MPTP assay. **(D)** Representative VDAC1, ANT1, and CYPD mRNA expression levels. ^a^ *p* < 0.05 compared with the CON group; ^aa^ *p* < 0.01 compared with the CON group; ^b^ *p* < 0.05 compared with the THP group; ^bb^ *p* < 0.01 compared with the THP group; ^c^ *p* < 0.05 compared with the IMQ group; ^cc^ *p* < 0.01 compared with the IMQ group; ^d^ *p* < 0.05 compared with the IQC+IMQ group; ^dd^ *p* < 0.01 compared with the IQC+IMQ group. Data were analysed using single-factor ANOVA followed by t-test. The data are expressed as the mean ± square error of the mean of three independent experiments.

Compared with the CON group, the mitochondrial activity and mitochondrial membrane potential of the THP group were significantly reduced. Compared with the THP group, IMQ inhibited mitochondrial activity and mitochondrial membrane potential. The mitochondrial activity and mitochondrial membrane potential of the cells in the IQC group increased significantly. Compared with the IMQ group, IQC+IMQ restored the mitochondrial activity and mitochondrial membrane potential. Compared with the IMQ+IQC group, the mitochondrial activity and mitochondrial membrane potential of the IQC group were significantly increased (Figures 6A, B).

The mitochondrial permeability transition refers to an increase in permeability in the inner mitochondrial membrane mediated by a channel, the MPTP. The calcein-AM/COCl_2_ method was used to detect the opening level of the MPTP in cardiomyocyte mitochondria (Figure 6C). The calcein-AM fluorescence intensity of the cells in the THP group was significantly lower than that in the CON group, indicating that the opening level of the MPTPs was significantly increased, that of the IMQ group was significantly reduced, and that of the IQC group was significantly increased. The calcein-AM fluorescence intensity of the cells in the IMQ+IQC group was significantly higher than that in the IMQ group. Compared with the IMQ+IQC group, the calcein-AM fluorescence intensity of the cells in the IQC group was significantly higher, and the opening level of MPTP was significantly reduced. These results suggested that IQC could inhibit the opening of the MPTPs in cardiomyocytes.

### 3.13 IQC improved the expression of mitochondrial membrane genes involved in THP-induced H9c2 and HCM cell damage

By detecting COX IV mRNA extracted from mitochondria within cells, the integrity of the extracted mitochondria is determined. The expression of COX IV mRNA in the extracted mitochondria of each group showed no significant difference, indicating that the mitochondria extracted in this study have good integrity (Supplementary Figure S1).

The expressions of the mitochondrial membrane genes VDAC1, ANT1, and CYPD in the THP group were significantly increased (Figure 6D). Compared with the THP group, the expression of mitochondrial membrane genes in the IMQ group was significantly increased, while IQC reversed the effect of THP, and the expressions of VDAC1, ANT1, and CYPD were significantly reduced. Compared with the IMQ group, the IMQ+IQC group showed reduced expression of the mitochondrial membrane genes. Compared with the IMQ+IQC group, the expressions of mitochondrial membrane genes in the IQC group were significantly reduced. These results indicated that IQC significantly reduced THP-induced mitochondrial damage. In addition, the AKT inhibitor IMQ partially blocked the effect of IQC on H9c2 and HCM cells with THP-induced cellular damage.

### 3.14 Prediction of drug targets of IQC against THP-induced cardiotoxicity

**FIGURE 7:**
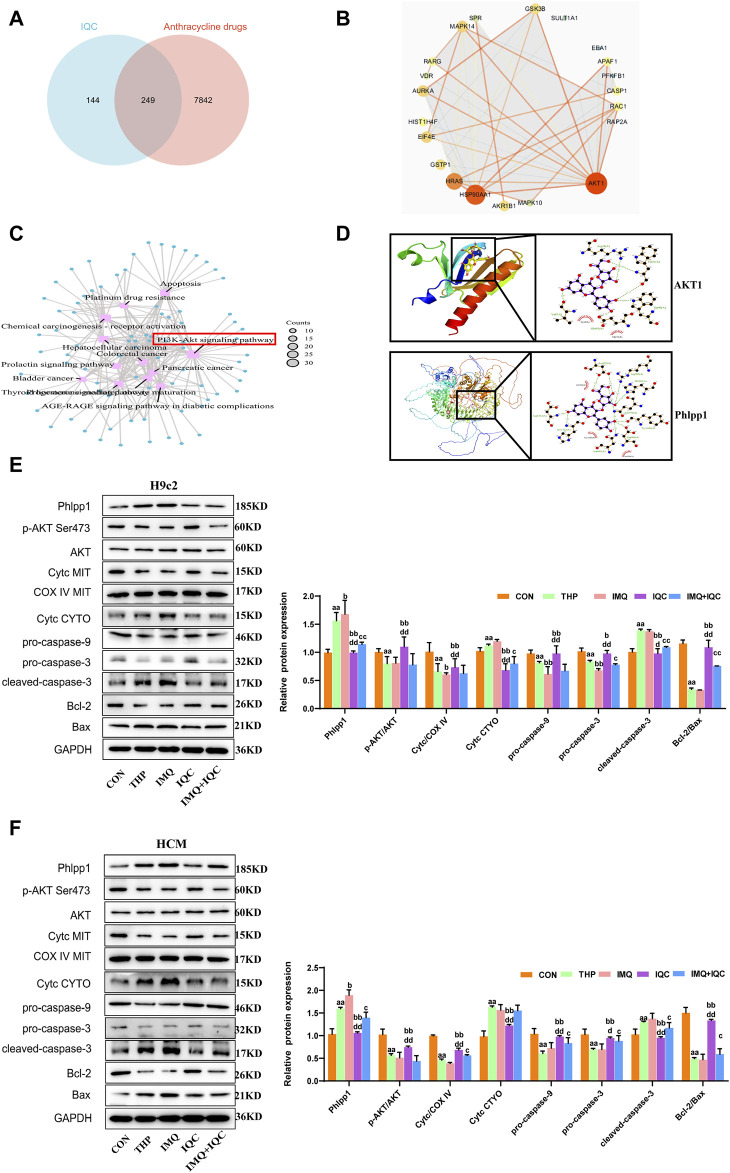
IQC reduced THP-induced cardiotoxicity by regulating the Phlpp1/AKT/Bcl-2 signalling pathway. **(A)** The 249 intersection targets of IQC against THP-induced cardiotoxicity (Venn diagram). **(B)** The PPI network of the top 21 intersection targets genes. **(C)** KEGG pathway enrichment analysis of the top 21 intersection targets genes. **(D)** The molecular docking model of IQC with Phlpp1 and AKT1. **(E,F)** Representative Phlpp1, Akt, Bcl-2, Bax, Cyt c and caspase protein expression levels in the cytoplasm or mitochondria; GAPDH or COX IV was used as an internal control. ^a^ *p* < 0.05 compared with the CON group; ^aa^ *p* < 0.01 compared with the CON group; ^b^ *p* < 0.05 compared with the THP group; ^bb^ *p* < 0.01 compared with the THP group; ^c^ *p* < 0.05 compared with the IMQ group; ^cc^ *p* < 0.01 compared with the IMQ group; ^d^ *p* < 0.05 compared with the IQC+IMQ group; ^dd^ *p* < 0.01 compared with the IQC+IMQ group. Data were analysed using single-factor ANOVA followed by t-test. The data are expressed as the mean ± square error of the mean of three independent experiments.

In order to delineate the mechanism underlying the anti-cardiotoxicity activity of IQC, we conducted network pharmacological analysis to predict the potential targets of IQC against THP-induced cardiotoxicity. We collected a total of 393 unique IQC-related targets, 8,091 specific pirarubicin cardiotoxicity-related targets, and finally identified 249 intersection targets by online Venn diagram (Figure 7A). Next, we constructed a protein-protein interaction (PPI) network to display the direct and indirect regulatory interactions among these intersection targets (Figure 7B). According to the presented PPI network, the top 21 hub protein targets were selected, and KEGG pathway enrichment analysis of the top 21 intersection targets genes (Figure 7C). We found that the PI3K-AKT signaling pathway is in there and may be an important target.

Then, we performed molecular docking simulation by AutoDock Vina to predict the possible binding of IQC to AKT1 and Phlpp1 (Figure 7D). The binding energy between IQC and AKT1 proteins is −6.3 kcal/mol, which proves to have a good binding effect. IQC interact with proteins, mainly through the formation of hydrogen bonds and hydrophobic forces, and hydrogen bonds are formed with Glu95, Trp99, Trp11, Arg41 and Val7. The lengths of hydrogen bonds are 2.72Å, 2.80Å, 2.87Å, 3.04Å, 2.95Å and 2.97Å, respectively. It has hydrophobic effects with Gly10 and Lys8.

The binding energy of IQC and Phlpp1 is −8.0 kcal/mol, which proves that it has a good binding effect. IQC interact with Phlpp1, mainly through the formation of hydrogen bonds and hydrophobic forces, with Asn992, Asp968, Tyr1409, Gln970, His926, Arg903, Asp923, Asp1413, His1412 Hydrogen bonds are formed. The lengths of hydrogen bonds are 3.45Å, 3.15Å, 3.05Å, 2.80Å, 3.29Å, 2.99Å, 2.98Å, 3.16Å, 2.78Å, 3.07Å and 3.12Å, respectively; with Leu946, Gly1410, Gly94 8 has a hydrophobic effect.

### 3.15 IQC targeting Phlpp1 to activate the AKT/Bcl-2 pathway in H9c2 and HCM cells

To further elucidate the mechanisms underlying the effect of IQC on THP-induced damage among cardiomyocytes, we detected mitochondrial and cytosolic levels of p-AKT, AKT, and Cyt c, as well as those of the caspase family, Bcl-2, Phlpp1and Bax protein expression in H9c2 and HCM cells (Figures 7E, F).

The expressions of p-AKT/AKT and mitochondrial Cyt c, pro-caspase-9, pro-caspase-3, and Bcl-2/Bax were significantly decreased, while the cytosolic levels of Phlpp1, Cyt c, and cleaved-caspase-3 were markedly increased by THP compared with the CON group. Compared with the THP group, IMQ significantly decreased the expression levels of p-AKT/AKT and mitochondria Cyt c, as well as those of pro-caspase-9, pro-caspase-3, and Bcl-2/Bax, and increased the levels of Phlpp1, cytosolic Cyt c, and cleaved-caspase-3. However, these results were improved by IQC. Compared with the IMQ group, p-AKT/AKT and Phlpp1 proteins in the IMQ+IQC group showed no significant changes. While p-AKT/AKT and mitochondrial Cyt c, pro-caspase-9, pro-caspase-3, and Bcl-2/Bax protein levels in the IMQ+IQC group were slightly higher, the levels of cytosolic Cyt c and cleaved-caspase-3 protein were lower. Compared with the IMQ+IQC group, the IQC group showed increased levels of p-AKT/AKT and mitochondrial Cyt c, pro-caspase-9, pro-caspase-3, and Bcl-2/Bax proteins, and reduced the expression levels of cytosolic Cyt c and cleaved-caspase-3. These results indicate that IQC alleviated THP-induced mitochondrial damage and apoptosis.

## 4 Discussion

THP belongs to the fourth-generation anthracycline family. Although anthracyclines increase the long-term survival of patients with cancer, they are associated with significant cardiovascular complications (Christiansen and Autschbach, 2006). THP-induced cardiotoxicity has been observed in animal studies (Wang et al., 2018; Wang H. et al., 2020). Wang et al. (2017) used THP to establish an H9c2 cell injury model and treated them with 5 μM of THP for 24 h. The results showed that the proliferation of H9c2 cells was inhibited, the survival rate was reduced, and the apoptotic rate increased significantly. In a study of the ability of miR-129-1-3p to minimise THP-induced damage of H9c2 cells, Li et al. (2020) also induced H9c2 cell damage using THP at a concentration of 5 μM for 24 h. In the present study, H9c2 and HCM cells were cultured *in vitro*, and THP was used to establish a cardiomyocyte injury model. The results showed that when the THP action time was short (6 and 12 h), low concentrations of THP (1, 3, and 5 μM) did not cause noticeable damage to the cells, compared to when the THP action time was too long (24 and 36 h) or the concentration was too high (7 and 9 μM). These values were too high to induce cell damage, and did not meet the experimental requirements; when THP was applied at a dose of 5 μM for 24 h, the survival rate was greater than 60%. This degree of damage is suitable for observing the effects of drugs on cardiomyocyte damage. The time and concentration of THP modelling are consistent with the experimental results of Wang and Li, indicating that this method can successfully replicate the THP-induced cardiomyocyte damage model *in vitro*.

The rodents’ body mass, hemodynamic parameters, electrocardiogram, and myocardial enzyme content were then measured. Our team has adopted a regimen of a single tail vein injection of 3 mg/kg THP once per week for 6 weeks, accurately replicating a rodent model of THP-induced myocardial damage. Rats ingested THP, causing them to lose weight and develop cardiac dysfunction. After intravenous injection of 3 mg/kg THP for 8 weeks, a series of systemic and cardiac toxicity changes were observed in SD rats, including abnormal body weight and food intake, adverse changes in echocardiography and electrocardiography readings, and cardiac tissue structure damage (Shi et al., 2021).

The natural substance IQC has been shown to have strong protective benefits against a variety of organ ailments (Valentová et al., 2014; Huang et al., 2018). Numerous studies have shown that THP-induced cardiotoxicity is linked to oxidative stress and apoptosis in cardiomyocytes (Dai et al., 2018; Park, 2020). When anthracyclines enter cardiomyocytes, they produce a large amount of ROS by reducing the redox cycle of the electron transport chain complex I, which in turn triggers an oxidative stress response (Davies et al., 1983). Moreover, anthracyclines have a strong affinity for cardiolipin, forming irreversible complexes through positive and negative charges, which accumulate on the inner mitochondrial membrane (Parker et al., 2001). When the accumulation of complexes in mitochondria exceeds 50 μM, it decreases the mitochondrial activity and alters the mitochondrial membrane potential. The results of the present study showed that IQC significantly reduced THP-induced H9c2 and HCM cellular oxidative stress, increased the mitochondrial activity and membrane potential, and improved mitochondrial membrane permeability.

The heart function of the rats was largely recovered and their body weight rose after receiving IQC therapy *in vivo*. Zwicker et al. (2019) discovered that in patients with advanced cancer, IQC improved indicators of coagulation by targeting extracellular protein disulfide isomerase. Cardiomyocyte structural alterations brought on by THP are considerably mitigated by IQC, as shown by HE findings. It has proven possible to identify myocardial necrosis with the help of cardiac markers such c-TnT, LDH, CK-MB, and BNP in clinical practice. Increased plasma BNP, CK-MB, CTnT, and LDH activities were seen after DOX treatment (Zhang Y. et al., 2020), indicating that the drug had induced intracellular space enlargement, cytoplasmic vacuolation, and myocardial cell abnormalities. His results are in line with our experimental studies that showed that IQC treatment reduced serum marker levels and reversed these abnormalities in heart. IQC reduced inflammation, oxidative stress, and heart cell apoptosis in a rat model of acute myocardial infarction (Ma et al., 2018). It also reduced the size of the infarct and levels of CK, CK-MB, and LDH. Experiments cited above demonstrate the beneficial effects of IQC in treating cardiovascular disorders.

The oxidative stress hypothesis and the molecular pathways it depends on are currently the mainstays of our understanding of the mechanism of anthracycline-induced cardiotoxicity (Davies et al., 1983). Heart tissue or cells have a poor antioxidant enzyme concentration, a low capacity to fight oxidation, and a heightened sensitivity to ROS. Researchers found that whereas THP increased MDA levels, it lowered SOD and GSH levels, suggesting that THP might lead to oxidative stress. Serum SOD levels were found to be considerably lower in the treatment group after pirarubicin administration (Wang et al., 2018). Our study found that IQC exhibited antioxidant properties, decreasing MDA and raising SOD and GSH levels at a dosage of 50–100 mg/kg or 70 µM. There is also strong evidence from many disease models demonstrating that IQC has antioxidant properties. Dai et al. (2018) suggested that IQC protected against oxidative stress and neuronal apoptosis in *in vivo* and *in vitro* cerebral I/R injury models via Nrf2-mediated inhibition of the NOX4/ROS/NF-κB signaling pathway.

Mitochondria serve crucial functions in regulating apoptosis. The depletion of intracellular ATP caused by dysfunctional mitochondria results in cell death (Vringer and Tait, 2023). Cardiolipin is a crucial part of the mitochondria’s inner membrane. Anthracyclines bind to cardiolipin and produce complexes that build up in the inner mitochondrial membrane, which reduces the activity of the mitochondria (Parker et al., 2001). Apoptotic signals cause the release of apoptotic signaling molecules, such as Cyt c, into the cytoplasm by decreasing mitochondrial membrane potential (Buttke and Sandstrom, 1994). VDAC1, ANT1, and CYPD are components of the composite channel known as MPTP, which is essential for apoptosis. CMXRos was used by Kong and Rabkin (2003) to mark embryonic chick cardiomyocytes that had received palmitate treatment. They claimed that red fluorescence in chick cardiomyocytes was less intense. This demonstrated that palmitate interfered with mitochondrial activities to cause apoptosis in chick cardiomyocytes. Samukelisiwe et al. used the JC-1 probe to mark changes in the mitochondrial membrane potential of DOX-induced H9c2 cells (Shabalala et al., 2019). In H9c2 cells, DOX made green fluorescence less intense while making red fluorescence more intense. In cardiac tissues of rats after heart damage, DOX enhanced the mRNA expression level of VDAC1, ANT1, and CYPD, but AVLE reversed this effect (Zhang et al., 2019). In this study, THP decreased Cyt c release, raised VDAC1, ANT1, and CYPD mRNA expression levels, and hindered mitochondrial activity and membrane potential. IQC treatment raised mitochondrial membrane potential, decreased VDAC1, ANT1, and CYPD mRNA expression levels, and prevented the release of Cyt c.

Cardiomyocyte and endothelial cells in cardiac tissues experience apoptosis when exposed to low doses of DOX (Hu et al., 2019; Zhang X. et al., 2020). In addition, the apoptosis of cardiomyocytes is greatly influenced by changes in mitochondrial function brought on by DOX. DOX promotes the transfer of Cyt c from the mitochondria to the cytoplasm. Apoptotic protease activator-1, ATP/dATP, and Caspase-9 zymogen join forces with cytochrome c to create an apoptotic complex in the cytoplasm. By activating Caspase-9, starting a cascade process driven by Caspase protein, and encouraging the cleavage of the effector enzyme Caspase-3, this complex causes cardiomyocyte apoptosis. DOX suppresses the AKT/Bcl-2 signaling pathway to cause apoptosis in the rat heart, according to Zhang et al. In order to cause apoptosis, DOX is said to be able to block the expression of the proteins p-AKT and Bcl-2/Bax, according to Bai and Wang (2019). Additionally, it has been shown that DOX can influence the release of cytochrome c directly or indirectly by increasing Bax levels and lowering Bcl-2 levels, which starts the apoptotic process. IQC treatment significantly decreased the levels of cytochrome c, cleaved caspase-9, and cleaved caspase-3 expression in cardiomyocytes, demonstrating that IQC treatment can lower cardiomyocyte apoptosis rates by preventing the activation of apoptotic pathways.

The Phlpp gene is found on chromosomes 18 and 16 in humans (Aviv and Kirshenbaum, 2010). Their physiological function is to specifically dephosphorylate phosphorylated AKT and inactivate the protein kinase activity, thereby inhibiting the effects of AKT (Newton and Trotman, 2014). Brognard and Newton found that when Phlpp1 is not expressed, agonists can induce a 30-fold increase in the phosphorylation level of AKT in cells when Phlpp1 is not expressed (Brognard and Newton, 2008). Activating the PI3K/AKT signaling pathway has been shown to ameliorate diabetic cardiomyopathy by inhibiting Phlpp1. In addition, according to a study, the lack of Phlpp1 can promote the proliferation of chondrocytes by increasing the expression of fibroblast growth factor 18 (Bradley et al., 2015). To investigate the function of AKT in shielding IQC against THP-induced cardiotoxicity, the Phlpp1/AKT/Bcl-2 signaling pathway was inhibited using the AKT inhibitor. IQC can bind to Phlpp1 and AKT, according to the molecular docking research. We show that THP elevates Phlpp1 and inhibits p-AKT in cardiomyocytes. p-AKT levels dropped, Phlpp1 levels rose, and cardiotoxicity worsened after administration of the AKT inhibitor. But IQC therapy reduced cardiotoxicity.

## 5 Conclusion

**FIGURE 8:**
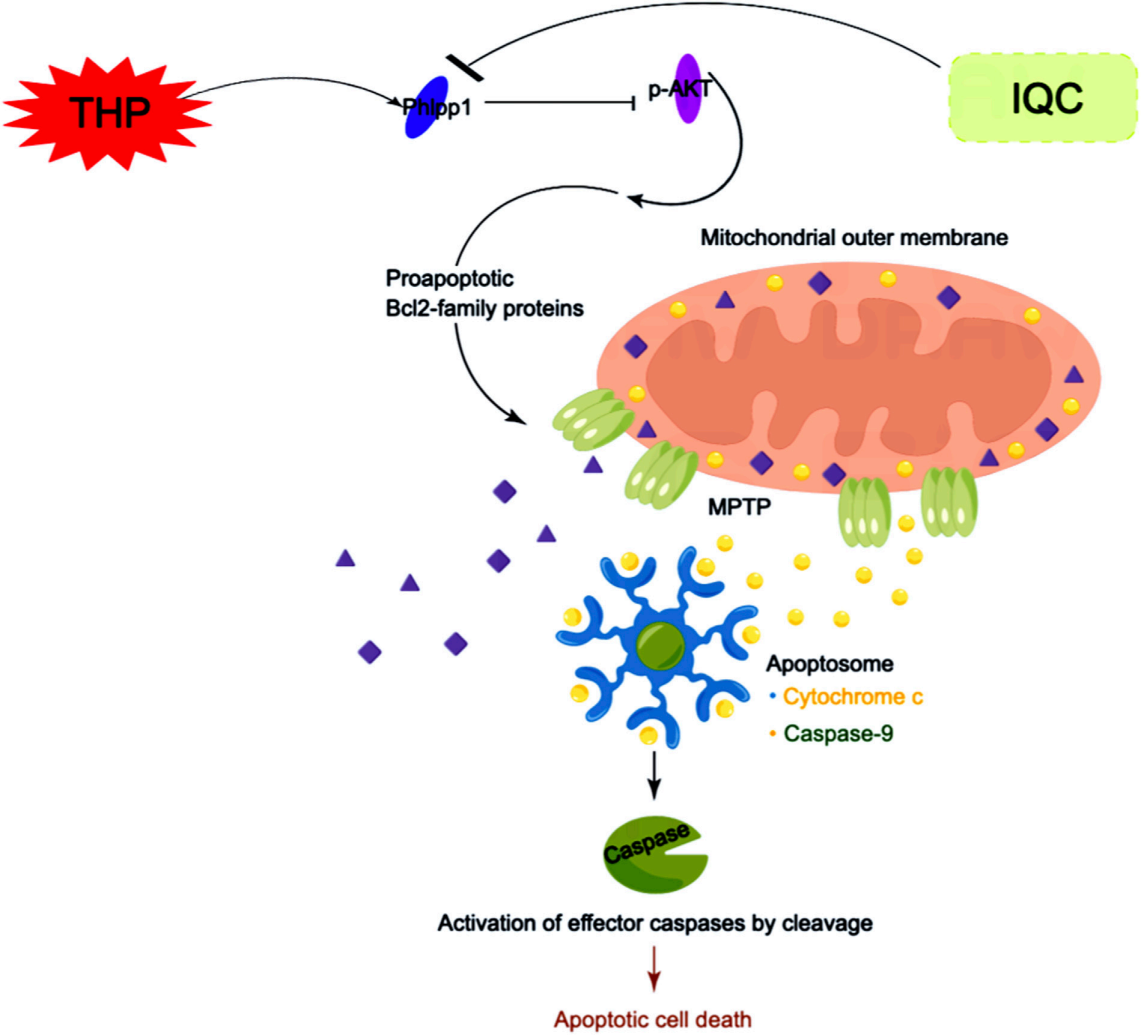
Scheme summarizes. IQC against THP-induced myocardial toxicity via Phlpp1/AKT/Bcl-2 signaling pathways.

Our results indicate that IQC protects the changes in mitochondrial membrane permeability in cardiomyocytes by regulating the Phlpp1/AKT/Bcl-2 signaling pathway, inhibits the release of cytc from the mitochondrial inner membrane to the cytoplasm, forms apoptotic bodies, induces cell apoptosis, and reduces THP induced cardiotoxicity (Figure 8).

## Data Availability

The datasets presented in this study can be found in online repositories. The names of the repository/repositories and accession number(s) can be found in the article/Supplementary Material.
