# Supplementary material for: Isoquercitrin alleviates pirarubicin-induced cardiotoxicity in vivo and in vitro by inhibiting apoptosis through Phlpp1/AKT/Bcl-2 signaling pathway
Source: Front Pharmacol. 2024 Mar 18;15:1315001. doi: 10.3389/fphar.2024.1315001 (PMC10982373; doi:10.3389/fphar.2024.1315001)
Supplement: Supplementary file 2 [file DataSheet2.docx]

**Isoquercitrin alleviates pirarubicin-induced cardiotoxicity *in vivo* and *in vitro* by inhibiting apoptosis through Phlpp1/AKT/Bcl-2 signaling pathway**

Lei Wang^a1^, Jiulong Ma^b1^, Chen Chen^b^, Bin Lin^c^, Sicong Xie^a^, Weiwei Yang^a^, Jiajia Qian^a^, Yang Zhang^abc^*

^a^ Department of Rehabilitation Medicine, School of Acupuncture-Moxibustion and Tuina and School of Health Preservation and Rehabilitation, Nanjing University of Chinese Medicine, Nanjing, China

^b^ Department of Experimental Pharmacology and Toxicology, School of Pharmacy, Jilin University, Jilin, China

^c^ Key Laboratory of Intelligent Pharmacy and Individualized Therapy of Huzhou; Department of Pharmacy, Changxing People's Hospital, Huzhou, China

* Corresponding author: Department of Rehabilitation Medicine, School of Acupuncture-Moxibustion and Tuina and School of Health Preservation and Rehabilitation, Nanjing University of Chinese Medicine, Nanjing, 210023, China.

E-mail addresses: yangzhang@njucm.edu.cn (Y. Zhang)

^1^ Co-First author.


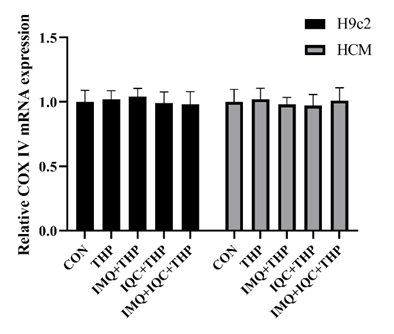


**Fig. S1** RT-PCR detection and extraction of mitochondrial integrity. Representative COX IV mRNA expression levels. ^a^ P<0.05 compared with the CON group; ^aa^ P<0.01 compared with the CON group; ^b^ P<0.05 compared with the THP group; ^bb^ P<0.01 compared with the THP group; ^c^ P<0.05 compared with the IMQ group; ^cc^ P<0.01 compared with the IMQ group; ^d^ P<0.05 compared with the IQC+IMQ group; ^dd^ P<0.01 compared with the IQC+IMQ group. Data were analysed using single-factor ANOVA followed by t-test. The data are expressed as the mean ± square error of the mean of three independent experiments.

**Fig. S2** THP reduces mitochondrial respiration in H9c2 and HCM cells, and IQC rescues mitochondrial respiration in THP-treated H9c2 and HCM cells. Mitochondrial energy metabolism was measured using a Seahorse Bioscience XFp analyzer. During testing, H9c2 cells were treated with 10 μM oligomycin, 10 μM FCCP, and 5 μM rotenone/antimycin A. (A) OCR image of H9c2 cells. (B) basal respiration, maximal respiration, ATP-associated OCR and proton leak of H9c2. (C) OCR image of HCM cells. (D) basal respiration, maximal respiration, ATP-associated OCR and proton leak of HCM. ^a^ P<0.05 compared with the CON group; ^aa^ P<0.01 compared with the CON group; ^b^ P<0.05 compared with the THP group; ^bb^ P<0.01 compared with the THP group; ^c^ P<0.05 compared with the IMQ group; ^cc^ P<0.01 compared with the IMQ group; ^d^ P<0.05 compared with the IQC+IMQ group; ^dd^ P<0.01 compared with the IQC+IMQ group. Data were analysed using single-factor ANOVA followed by t-test. The data are expressed as the mean ± square error of the mean of three independent experiments.
